# Effects of Aquatic‐Based vs Land‐Based Closed Kinetic Chain Exercises on Pain, Functional Performance, Knee Proprioception, Muscle Strength, and Kinesiophobia in Women Aged 40–70 Years With Knee Osteoarthritis: A Randomized Controlled Trial

**DOI:** 10.1002/hsr2.73032

**Published:** 2026-08-10

**Authors:** Mahshid Mohammadi, Farzaneh Gandomi, Parviz Soufivand, Shirin Assar

**Affiliations:** ^1^ Department of Sports Injuries and Corrective Exercises, Faculty of Sport Sciences Razi University Kermanshah Iran; ^2^ Rheumatology Department, Clinical Research Development Center, Imam Reza Hospital Kermanshah University of Medical Sciences Kermanshah Iran

**Keywords:** aquatic exercise, closed kinetic chain exercise, functional performance, kinesiophobia, Knee osteoarthritis, pain

## Abstract

**Background and Aims:**

Knee osteoarthritis (KOA) is a progressive degenerative disease causing pain, muscle weakness, and functional decline, particularly in postmenopausal women. While closed kinetic chain (CKC) exercises are effective for rehabilitation, the comparative efficacy of aquatic‐ versus land‐based environments for these exercises remains poorly understood. This study compared the effects of aquatic‐ and land‐based CKC exercises on pain, functional performance, knee proprioception, muscle strength, and kinesiophobia in women aged 40–70 years with KOA.

**Methods:**

Thirty women aged 40–70 years with knee osteoarthritis were randomly assigned to an aquatic CKC exercise group (*n* = 15) or a land‑based CKC exercise group (*n* = 15). Twenty‐nine participants completed the 8‐week intervention and were included in the final analysis. Both groups performed CKC exercises three times per week for 8 weeks. Primary outcomes were pain (visual analog scale) and functional performance (Western Ontario and McMaster Universities Osteoarthritis Index, Timed Up and Go, stair test, and 40‑meter walk). Secondary outcomes included self‑reported knee instability, kinesiophobia (Tampa Scale of Kinesiophobia), knee proprioception (Inclinometer), and isometric muscle strength assessed with a handheld dynamometer.

**Results:**

Both groups showed significant within‐group improvements in all measured variables (*p *< 0.05; aquatic *d* = 0.39 to 3.40; land *d* = 0.54 to 4.01). However, after adjusting for baseline values (ANCOVA), no significant between‐group differences were found in primary or secondary outcomes, except for the 40‐meter walk test, where the land‐based group showed significantly greater improvement (*p* = 0.03, *η*_*p*_^2^ =0.15).

**Conclusion:**

Both aquatic‐ and land‐based CKC exercises are equally effective in improving pain, functional capacity, and muscle strength in women with KOA. Clinicians can prescribe either environment based on patient preference, accessibility, and tolerance to weight‐bearing, as both yield comparable clinical benefits.

## 1 Introduction

Knee osteoarthritis (KOA) represents a significant global health challenge, with its detrimental impact on functional independence and patient quality of life. Furthermore, it is recognized as a leading cause of disability, accounting for approximately 3% of the total global disability burden [1]. It has been estimated that approximately 40% to 80% of individuals with radiographic features of knee osteoarthritis develop symptomatic disease [2]. Knee osteoarthritis commonly occurs among older adults and is associated with an increased risk of comorbidities, recurrent falls, and ultimately higher mortality [3]. Approximately 10%–30% of individuals with KOA experience severe pain and functional limitations; thus, the disability rate is gradually increasing [3]. Osteoarthritis management is generally classified into pharmacological, non‐pharmacological, and surgical approaches. Among non‐pharmacological methods, exercise therapy has been shown to be an effective, low‐cost, and sustainable option for improving physical activity, muscle strength, flexibility, and range of motion [4]. Systematic reviews and clinical practice guidelines strongly recommend implementing targeted therapeutic exercise programs and education to improve pain management and functional abilities in individuals with hip and KOA [2, 5, 6].

The category of the chosen exercise plays a vital role in optimizing the potential health‐related benefits, offering unique advantages based on a patient's objectives and physical condition. Aquatic‐based exercises, performed in the supportive environment of pools, have become increasingly favored by KOA patients and healthcare practitioners as a gentle alternative. This preference is attributed to buoyancy and hydrostatic pressure, which support body weight, reduce joint loading, and diminish pain. Furthermore, the warmth of the water helps relax muscles, reduces stiffness, and facilitates movement [7, 8]. However, many patients cannot participate in aquatic interventions due to barriers such as rheumatoid arthritis, cold weather, higher costs, or fear of falling. Therefore, finding an alternative land‐based exercise method with comparable results could offer a viable option for these patients. Therefore, finding an alternative land‐based exercise method with similar results to aquatic exercise intervention could offer hope to patients facing difficulties with aquatic exercise. In their systematic review of the differences between land and water exercises for patients with knee osteoarthritis, Gibson et al. reported that due to the limited number of studies and their slight differences, it was not possible to draw definitive conclusions from their findings [9]. A meta‐analysis by Batterham found no differences in outcomes between aquatic and land‐based rehabilitation strategies. Most trials had design flaws, limiting confidence in observed effects [10]. Consequently, implementing an exercise regimen to improve joint proprioception may help alleviate KOA symptoms. Given that previous review evidence has supported CKC exercises for short‐term pain reductions and physical function improvement among individuals with KOA, this modality was selected as the intervention for this study [11].

Closed kinetic chain exercises involve multi‐joint movements performed under weight‐bearing conditions, simultaneously engaging several muscle groups, including agonists and antagonists [12]. It is suggested that CKC exercises are safer than open kinetic chain, as the forces generated are less likely to cause tissue damage [13]. In recent years, CKC exercises have gained significant popularity in clinical knee rehabilitation [14]. These exercises enhance proprioception and physical function, which are crucial for dynamic joint stabilization [15]. Furthermore, CKC exercise is widely recognized for its ability to increase muscle strength and enhance proprioception through the stimulation of muscle spindles and joint mechanoreceptors. By mimicking real‐world functional movements such as walking, climbing stairs, and standing up from a chair these exercises effectively prepare patients to perform daily activities with greater stability [1].

Despite the growing interest in exercise‐based rehabilitation for KOA, limited evidence exists regarding the comparative effectiveness of CKC exercises performed in different environments. In particular, few studies have investigated whether an aquatic environment offers additional benefits compared with land‐based training for patients experiencing KOA. While aquatic exercise may reduce joint loading through buoyancy and hydrostatic pressure, whereas land‐based exercise may provide greater mechanical stimulation of muscles and joint mechanoreceptors. Therefore, the present randomized controlled trial aimed to compare the effects of aquatic and land‐based CKC exercise programs on pain, functional performance, knee instability, proprioception, kinesiophobia, and muscle strength in women with KOA. It was hypothesized that CKC exercises performed in a land environment would produce comparable decrease in pain and improvement in functional outcomes to those performed in an aquatic environment.

## 2 Materials and Methods

### 2.1 Participants

This randomized controlled trial was conducted at Razi University, Kermanshah, Iran. Participants were recruited through local rehabilitation centers and orthopedic clinics from January 1, 2024, to April 12, 2024. Women aged 40–70 years with clinically diagnosed knee osteoarthritis according to the American College of Rheumatology criteria [16], confirmation of mild or moderate KOA severity with a Kellgren‐Lawrence classification based on radiographic evidence from a knee radiograph conducted within the last 6 months [17] were screened for eligibility. Inclusion criteria were: (1) presence of knee pain for at least 3 months prior to enrollment, (2) self‐reported knee instability during daily activities, and (3) ability to participate in an exercise program. Exclusion criteria included: (1) history of knee surgery in the past year, (2) inflammatory joint diseases, (3) neurological disorders affecting balance or movement, (4) severe cardiovascular disease, (5) uncontrolled hypertension (6), or (7) participation in structured lower‐limb rehabilitation programs within the previous 3 months [18, 19]. All participants reported knee joint pain associated with osteoarthritis. Individuals with severe pain in other lower extremity joints (such as hip or ankle) that could interfere with exercise performance were excluded from the study. Participants were instructed not to change their usual level of physical activity during the study period. Most participants had a low to moderate physical activity level and were not involved in regular structured exercise programs prior to the study. All the patients received the same drug regimens, which consisted of meloxicam (7.5 mg), glucosamine sulfate (750 mg), and Calcium‐D (Calcium elemental (500 mg) + Calcium carbonate (1250 mg) + Vitamin D3 (200 IU)) on a daily basis.

Patients were randomized into two groups using Random Allocation Software version 2.0.0. (Block size = 6) Before the intervention, all participants underwent an initial evaluation by the outcome assessor, who recorded each patient's name and identification number in a sealed envelope. After baseline outcome measures were taken, the envelope was opened, and the patients were assigned to one of two groups: aquatic CKC or land‐based CKC. The assessor and the person responsible for implementing the interventions were two different individuals. This setup ensured the person conducting the assessments was unaware of the patient's assigned group. However, it was not possible to blind the patients.

**Figure 1:**
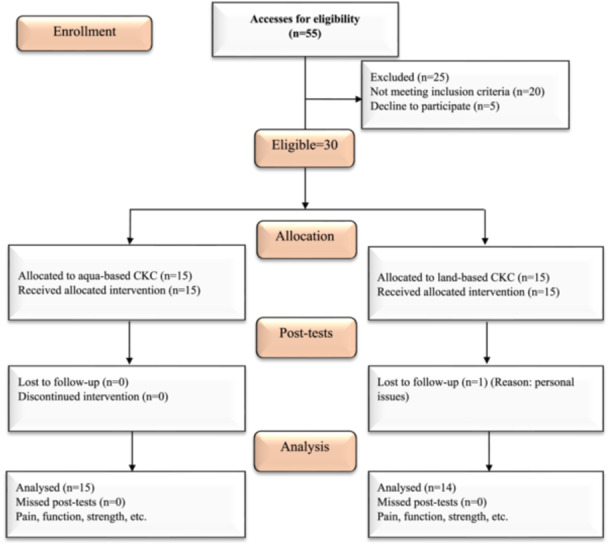
CONSORT flow diagram showing the participant allocation and follow‐up in land‐based and aquatic CKC exercise groups.

The sample size was calculated using G*Power® (Version 3.1.9.6). Test family: F test, statistical test: one‐way analysis of covariance (ANCOVA) using baseline measures as a covariate (one covariate = pretests). The goal was to detect an effect size of 0.59 [16], 80% power (1‐ß), a two‐sided significance level (α) of 0.05, and degrees of freedom (df) = 1, 26 participants (13 per arm) were determined. To account for a 10% anticipated loss to follow‐up (*n* = 3 out of 30), the total sample size was increased to 30 participants (15 per arm) (Figure 1).

### 2.2 Interventions

**Figure 2:**
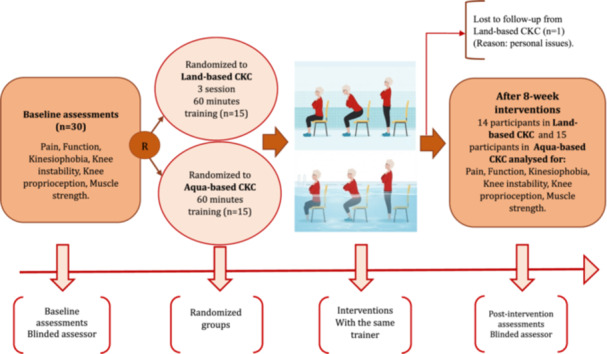
Schematic representation of the study protocol. The diagram illustrates the timeline for baseline and post‐intervention assessments (at 8 weeks) for both land‐based and aquatic CKC exercise groups. CKC, Closed Kinetic Chain.

The participants were randomly assigned to the aquatic CKC and land‑based CKC training groups (Figure 2). Both groups followed an identical exercise protocol in their respective environments. The intervention lasted 8 weeks, with three supervised sessions per week. To ensure consistency and safety, all sessions were conducted under the supervision of two corrective exercise specialists and overseen by a physiotherapist. Each 60‐min session was divided into three phases: a). a 10‐min warm‐up (walking (forward, backward, and side to side) and static stretching), b). 40 min of the specific CKC exercise protocol, and c). a 10‐min cool‐down (static stretching and breathing exercises). For the aquatic group, the exercises were performed in the shallow area of a pool (depth of 95 cm) with the water level at approximately the xiphoid process to reduce joint loading while maintaining a weight‐bearing stimulus. The pool temperature was maintained at a thermoneutral 32°C.

The CKC exercise protocol was progressively structured over 8 weeks, as detailed in Appendix 1. The exercises included quadriceps setting, heel raises, mini‐squats (30–45°), step‐ups/downs, wall slides, terminal knee extension, and various type of lunges. Resistance was progressed according to the overload principle using equipment such as resistance bands, weights, and Pilates balls [18, 19]. Training volume was systematically increased: sessions began with 3 sets of 10 repetitions in the first 3 weeks and gradually progressed to 3 sets of 15 repetitions by the final 2 weeks (Appendix 1). Exercise intensity was monitored using the Borg Rating of Perceived Exertion scale (RPE; 6 = no exertion, 20 = maximal exertion with a target range of 13–15 (somewhat hard) [20]. A 60‐second rest period was provided between sets to ensure adequate recovery and maintenance of proper exercise form.

### 2.3 Outcome Measures

#### 2.3.1 Primary Outcomes (Pain, Function)

Pain severity was assessed using the Visual Analogue Scale (VAS), a simple, reliable, and easy‐to‐administer instrument with excellent reliability (ICC = 0.97) [21]. The VAS consists of a 10‐cm horizontal or vertical line anchored by two extremes: “no pain” at 0 and “worst imaginable pain” at 10. Participants were instructed to indicate their perceived pain intensity by marking a point on the line. Pain severity was then quantified by measuring the distance from the “no pain” anchor to the participant's mark.

The Western Ontario and McMaster Universities Osteoarthritis Index (WOMAC) is a disease‐specific self‐report questionnaire to assess functional ability in hip or knee osteoarthritis patients. The WOMAC scale is a five‐item Likert questionnaire, where the severity of symptoms is graded from 0 to 4, with the following categories: none (0), mild (1), moderate (2), severe (3), and very severe (4). Patients select the category that best represents the severity of their symptoms. The WOMAC scale includes three subscales: pain, function, and joint stiffness (ICC = 0.87, ICC = 0.98). Scores range from 0 to 96, with lower scores indicating better status (less pain, stiffness, and functional limitation) [22].

The functional capacity of participants was assessed using the Timed Up and Go (TUG) test. It involves standing up from a chair, walking three meters, turning around, and returning to sit in the chair. The total time taken (in seconds) serves as the performance metric (ICC = 0.95–0.97) [23].

Additionally, the time to ascend and descend, eight standard steps, each 16 cm in height, at a comfortable speed, was recorded by a Chronometer, and the total time taken was recorded. A lower recorded time indicated better performance [24].

For the 40‐meter walking test, patients were instructed to walk the 40‐meter distance as fast as possible, and the total time taken was recorded. A lower recorded time indicated better performance [24].

#### 2.3.2 Secondary Outcomes (Self‐Reported Knee Instability, Kinesiophobia, Knee Proprioception, Isometric Muscle Strength)

Knee instability was assessed by asking participants about any instances of buckling, shifting, or giving way in their knee within the last week. Participants were asked to rate how much these issues affected their daily activities on a 6‐point numerical scale (0 = The symptom prevents me from all daily activities, 1= The symptom severely affects my activity, 2 = The symptom moderately affects my activity, 3 = The symptom slightly affects my activity, 4 = I have the symptom but it does not affect my activity, 5 = I do not experience giving way, buckling, or shifting of the knee [25]. Their responses provided insight into the level of knee instability.

The Tampa Scale of Kinesiophobia (TSK) is a 17‐item questionnaire to assess the degree of fear of movement. Each item is scored on a four‐point Likert scale, ranging from “strongly disagree” to “strongly agree,” with a maximum possible total score of 68. A higher TSK score indicates a more severe level of kinesiophobia. The scale demonstrates high internal consistency and construct validity [26].

Knee proprioception in patients was evaluated by measuring target angle reconstruction error. To assess joint proprioception, patients were asked to actively locate the knee position that had been passively taught to them. The Baseline Bubble Inclinometer (FS622, USA) was used for this assessment. The knee was gradually moved from 90° flexion to passive extension, with pauses of 10 s at 70° and 20° extension angles, during which the patients were instructed on these positions. Following this, the knee was returned to 90°, and the patients were asked to identify the previously taught angles with their eyes closed. They attempted to replicate these angles by moving their knees from the starting position to the taught 70° and 20° extension angles. Both the target angles and the angles identified by the patients were recorded. The mean reconstruction error was calculated based on three repetitions at each angle [27].

The isometric strength of the knee flexors (ICC = 0.83) [28], knee extensors (ICC = 0.95) [28], hip extensors (ICC = 0.97) [29], and hip abductors (ICC = 0.97) [29] was assessed using a hand‐held Baseline Electronic Push/Pull Dynamometer (ICC = 0.78–0.95) [30] manufactured by NY, USA (Model: 12‐0341). These muscle groups were selected because they are commonly affected in individuals with knee osteoarthritis.

For assessment of knee extensors strength, participants were seated at the edge of the examination table with the hip and knee flexed to 90°. The pelvis was stabilized using a nonelastic strap to minimize compensatory movements. The dynamometer was positioned on the anterior aspect of the leg just proximal to the medial malleolus, and resistance was applied against knee extension. The maximum force generated was recorded as quadriceps isometric strength.

To evaluate knee flexors strength, participants were positioned prone while the dynamometer was placed on the posterior aspect of the leg above the Achilles tendon. Resistance was applied against knee flexion, and the peak force produced was recorded as knee flexors isometric strength.

Hip abductors strength was measured in the side‐lying position. The tested limb was abducted away from the body midline while the trunk was stabilized. The dynamometer was placed approximately 5 cm above the lateral femoral condyle, and maximal isometric force was recorded against manual resistance.

For hip extensors strength assessment, participants were positioned prone with the knee flexed between 60° and 90°. The dynamometer was placed on the posterior aspect of the thigh approximately 2 cm above the popliteal fossa, and resistance was applied during hip extension to record maximal isometric strength. For all muscle groups, participants performed maximal voluntary isometric contractions for 3 s, and the highest recorded force was used for analysis.

### 2.4 Statistical Analysis

Statistical analysis was performed using SPSS version 27.0 (IBM Corp., Armonk, NY, USA). The normality of data distribution and homogeneity of variances were confirmed using the Shapiro–Wilk and Levene's tests, respectively. As the data satisfied the assumptions for parametric inference, descriptive statistics are reported as means ± standard deviations (SDs) with 95% confidence intervals (CIs).

The primary analysis followed a prespecified approach to evaluate the efficacy of the interventions. Specifically, one‐way Analysis of Covariance (ANCOVA) was conducted to compare between‐group differences in post‐test scores, utilizing baseline (pre‐test) values as a covariate to improve the precision of the estimates. For within‐group comparisons (pre‐test to post‐test), paired‐sample t‐tests were utilized.

The magnitude of treatment effects was quantified using Effect Sizes; Partial Eta Squared (*η*_*p*_^2^) was employed for ANCOVA and interpreted as small (0.01–059), medium (0.06–0.139), or large (≥ 14). Cohen's *d* was used for within‐group changes and interpreted as small (0.20–0.49), medium (0.50–0.79), or large (≥ 0.80) based on standard criteria [31]. Statistical reporting was guided by the recommendations of Assel et al. (2018) and Lang and Altman (2013) to ensure the precision and transparency of the findings. The significance level for all two‐tailed tests was set at α = 0.05 [32, 33].

The minimal detectable change at the 95% confidence level (MDC_%95_) was calculated using the standard error of measurement (SEM). SEM was computed from the baseline standard deviation (SD_baseline_) and the test–retest reliability coefficient (r) as:

$$\text{SEM}={\text{SD}}_{\text{baseline}}\times \surd \left(1-r\right)$$

$${\text{MDC}}_{\%95}=1.96\times \surd 2\times \text{SEM}$$

The minimal clinically important difference (MCID) was estimated using a distribution‐based approach based on the reliable change index (RCI). The MCID threshold based on RCI was calculated as:

MCID_RCI_ = 1.96 × SD_baseline_ × √(2 × (1 − r))

We prespecified that MCID values would be considered acceptable when MCID ≥ MDC_%95_, and clinical improvement would be interpreted when the mean change score (Δ) exceeded the MCID threshold [34].

### 2.5 Ethical Considerations

The study protocol was reviewed and approved by the Research Ethics Committee of Razi University (registration No. IR.RAZI.REC.1402.37). The research has also been registered in the Iranian Registry of Clinical Trials (code: IRCT20230409057863N1, https://irct.behdasht.gov.ir/user/trial/69519/view, registered 2023. 12. 20; The recruitment for this study was lasted from 2024. 01. 01 to 2024. 04. 12). In addition, written informed consent was obtained from the participants.

## 3 Results

A total of 30 of the 55 screened patients were enrolled in the study. Out of 30 randomized participants, 29 (96.6%) completed the study, with a 3.3% attrition rate (n = 1/30) in the land‐based group due to personal reasons. The study flowchart is shown in Figure 1. There were no statistically significant differences between the groups at baseline. The descriptive characteristics and baseline results of the participants are presented in Table 1. Preliminary statistical checks confirmed that the majority of outcome measures followed a normal distribution (Shapiro–Wilk test, *p *> 0.05) and met the assumption of homogeneity of variances (Levene's test, *p *> 0.05). Accordingly, parametric tests were utilized for further analyses, and results are reported as Mean ± SD with 95% confidence intervals.

**Table 1:** Baseline demographic and clinical characteristics of participants.

Study parameters	LCKC (*n* = 14)	ACKC (*n* = 15)	*p‐value
	M (SD)	M (SD)	
Age (yrs.)	55.85 (9.53)	54.66 (8.45)	0.72
Weight (Kg)	74.00 (8.66)	80.00 (10.28)	0.10
Height (cm)	160.00 (4.80)	161.46 (8.20)	0.56
BMI (kg/m^2^)	28.82 (2.21)	30.82 (4.66)	0.15
Pain (0‐10)	5.71 (1.98)	6.80 (1.97)	0.14
WOMAC OA index (0‐96)	55.21 (8.95)	56.00 (14.84)	0.86
TUG (s)	14.25 (2.97)	14.40 (2.71)	0.88
40‐m walking time (s)	43.58 (4.59)	46.44 (7.86)	0.24
8‐step stairs up walking time (s)	6.86 (3.73)	6.70 (2.87)	0.88
8‐step stairs down walking time (s)	5.76 (4.57)	5.82 (2.76)	0.96
Kinesiophobia (0‐68 points)	47.07 (5.35)	49.73 (6.43)	0.23
Knee extension proprioception at 20 degrees	8.92 (4.35)	7.73 (4.62)	0.48
Knee extension proprioception at 70 degrees	7.63 (7.12)	5.64 (4.04)	0.35
Knee instability score (0‐5)	1.85 (0.66)	1.53 (0.63)	0.19
Knee extensors strength (kg)	20.89 (5.09)	23.61 (8.17)	0.29
Hip extensors strength (kg)	14.45 (3.69)	16.99 (6.41)	0.20
Hip abductors strength (kg)	15.20 (4.80)	16.97 (6.33)	0.040
Knee flexors strength (kg)	12.12 (2.83)	12.81 (4.23)	0.61

Abbreviations: ACKC, aqua‐based close kinetic chain; BMI, body mass index; LCKC, land‐based close kinetic chain; M (SD), mean (standard deviation); s, second; TUG, timed up‐and‐go; WOMAC, Western Ontario and McMaster Universities Arthritis Index; Yrs., years.

*p‐values were derived from independent t‐tests to compare baseline characteristics between groups.

**Figure 3:**
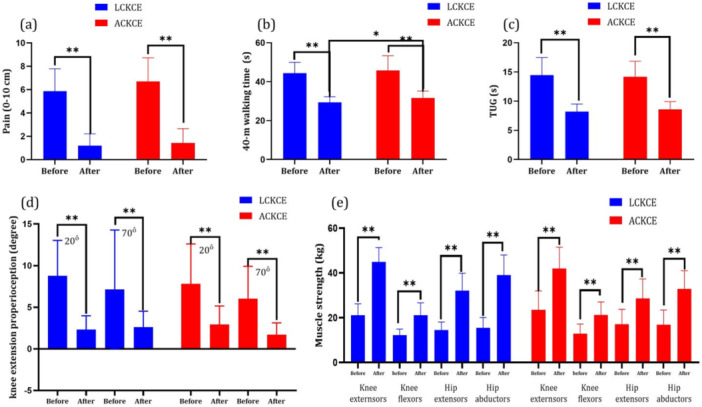
Bfore and after outcomes in the land‐based and aquatic CKC groups. Mean (SD) values are shown for (a) pain intensity, (b) 40‐m walking time, (c) Timed up‐and‐go (TUG) test, (d) knee extension proprioception at 20° and 70°, and (e) muscle strength. LCKCE, land‐based closed kinetic chain exercises; ACKCE, aquatic closed kinetic chain exercises. *p < 0.05, **p < 0.01 (within‐group and between group comparisons).

Table 2 and Figure 3 present the results of the paired‐samples t‐tests comparing pre‐ and post‐intervention scores within each group. Statistically significant improvements were observed in both groups across most measures (all *p *< 0.05). In the land‐based CKC group, effect sizes ranged from small to large (Cohen's *d* = 0.52 to 4.01), and similar improvements were noted in the aquatic CKC group (Cohen's *d* = 0.38 to 3.40).

**Table 2:** Within‐group changes (Pre‐ to Post‐intervention) in study variables.

Study parameters	LCKC group (*n* = 14)				ACKC group (*n* = 15)			
	Mean (SD)	Difference (95% CI)	*p*‐value	*Cohen's d*	Mean (SD)	Difference (95% CI)	*p*‐value	*Cohen's d*
Pain (0–10)	4.42 (1.78)	(3.39, 5.45)	**< 0.001**	0.57	5.46 (2.17)	(4.11, 6.82)	**< 0.001**	0.57
WOMAC OA index (0–96)	37.35 (12.90)	(29.90, 44.80)	**< 0.001**	0.60	40.93 (17.38)	(31.30, 50.55)	**< 0.001**	0.56
TUG time (s)	6.15 (2.40)	(4.76, 7.54)	**< 0.001**	0.57	5.70 (2.37)	(4.37, 7.01)	**< 0.001**	0.57
40‐m walking time (s)	14.55 (4.78)	(11.78, 17.31)	**< 0.001**	0.61	14.54 (7.57)	(10.34, 18.74)	**< 0.001**	0.61
8‐step stairs up walking time (s)	4.45 (2.79)	(2.8, 6.07)	**< 0.001**	0.45	4.07 (3.10)	(2.36, 5.79)	**< 0.001**	0.45
8‐step stairs down walking time (s)	3.67 (3.83)	(1.45, 5.88)	**< 0.001**	0.33	3.39 (2.93)	1.76, 5.01)	**< 0.001**	0.33
Kinesiophobia (0–68 points)	8.21 (7.40)	(3.94, 12.48)	**0.001**	0.37	12.13 (8.14)	(7.62, 16.64)	**< 0.001**	0.45
Knee extension proprioception at 20 degrees	6.54 (4.95)	(3.69, 9.40)	**< 0.001**	0.41	4.89 (4.01)	(2.66, 7.11)	**< 0.001**	0.41
Knee extension proprioception at 70 degrees	4.95 (7.13)	(0.83, 9.07)	**0.02**	0.27	3.93 (4.65)	(1.35, 6.50)	**0.006**	0.27
Knee instability score (0–5)	−2.92 (0.73)	(−3.35, −2.50)	**< 0.001**	1.87	−3.20 (0.94)	(−3.72, −2.67)	**< 0.001**	2.13
Knee extensors strength (kg)	−23.77 (7.78)	(−28.26, −19.27)	**< 0.001**	0.61	−18.72 (10.51)	(−24.55, −12.90)	**< 0.001**	0.61
Hip extensors strength (kg)	−17.60 (8.47)	(−22.49, −12.71)	**< 0.001**	0.52	−11.88 (10.19)	(−17.50, −6.26)	**< 0.001**	0.52
Hip abductors strength (kg)	−23.66 (9.91)	(−29.38, −17.93)	**< 0.001**	0.56	−16.47 (9.84)	(−21.92, −11.02)	**< 0.001**	0.54
Knee flexors strength (kg)	−9.01 (5.91)	(−12.43, −5.60)	**< 0.001**	0.44	−8.43 (4.96)	(−11.18, −5.68)	**< 0.001**	0.44

*Note:* Within‐group comparisons were performed using paired sample t‐tests. Mean (SD) values are presented.

Abbreviations: ACKC, aquatic closed kinetic chain; CI, confidence interval; LCKC, land‐based closed kinetic chain; SD, standard deviation; TUG, timed up‐and‐go; WOMAC, The Western Ontario and McMaster Universities Arthritis Index.

Table 3 displays the results of the ANCOVA, comparing outcomes between the two groups after adjusting for baseline values. There were no statistically significant between‐group differences for most outcomes. However, the land‐based CKC group demonstrated a significantly greater improvement in the 40‐meter walking test compared to the aquatic CKC group (*p* = 0.03, *η*_*p*_^2^ = 0.15), with a 95% CI of [−4.83 to −0.17].

**Table 3:** Between‐group differences in study variables adjusted for baseline (ANCOVA).

Study parameters	LCKC group (*n* = 14)	ACKC group (*n* = 15)	Mean difference (95% CI)	F	*p*‐value	*η*_*p*_^2^
	Mean (SD)	Mean (SD)				
Pain (0–10)	1.31 (0.99)	1.30 (1.22)	(−0.94, 0.84)	0.00	0.98	0.00
WOMAC OA index (0–96)	17.89 (12.46)	15.03 (8.40)	(−5.31, 11.03)	0.51	0.47	0.02
TUG time (s)	8.11 (1.22)	8.68 (1.32)	(−1.39, 0.26)	1.96	0.17	0.07
40‐m walking time (s)	29.20 (2.43)	31.72 (3.50)	(−4.83, −0.17)	4.86	**0.03**	0.15
8‐step stairs up walking time (s)	2.41 (0.45)	2.67 (0.54)	(−0.59, 0.16)	1.33	0.25	0.04
8‐step stairs down walking time (s)	2.09 (0.36)	2.42 (0.60)	(−0.72, 0.05)	3.16	0.08	0.10
Kinesiophobia (0–68 points)	38.87 (5.00)	37.58 (5.27)	(−2.81, 5.40)	0.41	0.52	0.01
Knee extension proprioception at 20 degrees	2.31 (1.69)	2.89 (2.16)	(−2.07, 0.91)	0.63	0.43	0.02
Knee extension proprioception at 70 degrees	2.68 (1.95)	1.70 (1.36)	(−0.34, 2.30)	2.30	0.14	0.08
Knee instability score (0–5)	4.80 (0.42)	4.71 (0.45)	(−0.26, 0.44)	0.27	0.60	0.01
Knee extensors strength (kg)	45.07 (6.69)	41.96 (9.31)	(−3.18, 9.40)	1.03	0.31	0.03
Hip extensors strength (kg)	32.25 (8.03)	28.69 (8.46)	(−3.09, 10.15)	1.22	0.27	0.04
Hip abductors strength (kg)	39.07 (9.23)	33.30 (8.22)	(−1.12, 12.55)	2.94	0.09	0.10
Knee flexors strength (kg)	21.35 (5.77)	21.04 (5.58)	(−3.79, 4.41)	0.02	0.87	0.00

*Note:* Mean (SD) values are adjusted for baseline scores. Comparisons were performed using one‐way ANCOVA.

Abbreviations: ACKC, aqua‐based closed kinetic chain; ANCOVA, analysis of covariance; CI, confidence interval; LCKC, land‐based closed kinetic chain; SD, standard deviations; TUG, timed up‐and‐go; WOMAC, Western Ontario and McMaster Universities Arthritis Index; *η*_*p*_^*2*^, partial eta squared (effect size).

The clinical significance of the improvements was assessed by comparing the mean change scores (Δ scores) of each group against the MDC and MCID (Table 4). In both the land‐based CKC and aquatic CKC groups, the Δ scores for all study variables exceeded their respective MDC and MCID values, indicating that the observed improvements were both statistically significant and clinically meaningful.

**Table 4:** The MDC and MCID values of the study variables.

Study parameters	SD_Baseline_ intervention groups	∆ Score of ACKC	∆ Score of LCKC	SEM	MDC_%95_	MCID (RCI)
Pain (0–10 cm)	1.88	**5.24**	4.43	0.56	1.54	1.57
WOMAC OA index (0–96)	12.14	**40.94**	37.36	1.71	4.74	4.75
TUG time (s)	2.79	5.70	**6.16**	0.05	0.16	1.85
40‐m walking time (s)	6.54	14.54	**14.55**	3.13	6.23	8.5
8‐step stairs up walking time (s)	2.76	4.07	**4.46**	0.87	2.40	2.41
8‐step stairs down walking time (s)	3.39	3.40	**3.67**	1.05	2.90	2.92
Kinesiophobia (0–68 points)	5.98	**12.13**	8.22	1.19	5.22	5.24
Knee extension proprioception at 20 degrees	4.46	4.89	**6.55**	2.23	6.16	6.18
Knee extension proprioception at 70 degrees	5.72	3.94	**4.95**	2.86	7.90	7.92
Knee instability score (0–5)	0.66	**3.20**	2.90	0.33	0.91	0.92
Knee extensors strength (kg)	6.88	18.73	**23.78**	1.53	4.18	4.26
Hip extensors strength (kg)	5.34	11.88	**17.61**	1.17	3.21	3.24
Hip abductors strength (kg)	5.62	16.47	**23.67**	1.23	3.41	3.48
Knee flexors strength (kg)	3.58	8.43	**9.02**	0.78	2.17	2.21

*Note:* Bold values indicate a numerically greater change score (Δ Score) in the respective intervention group compared to the other.

Abbreviations: ACKC, aqua‐based closed kinetic chain; LCKC, land‐based closed kinetic chain; MCID, minimal clinically important difference; MDC_%95_, minimal detectable change at the 95% confidence level; RCI, reliable change index; SEM, standard error of measurement; SD, Standard deviation; TUG, timed up‐and‐go; Δ score, change in mean (pretest ‐ posttest).

Specifically, the land‐based CKC group demonstrated larger Δ scores in functional tasks including TUG, 40‐meter walking, and stair negotiation (both up and down), as well as in knee proprioception and all muscle strength measures. Conversely, the aquatic CKC group showed greater clinical improvements in pain reduction, WOMAC function, kinesiophobia, and KI compared to the land‐based CKC group (Table 4).

## 4 Discussion

The present study investigated the effects of aquatic and land‐based CKC exercises on several clinical outcomes in women with knee osteoarthritis and knee instability, including pain, functional capacity, proprioception, muscle strength, and kinesiophobia. The findings of the present study showed no significant difference between aquatic and land‐based CKC exercises regarding pain, function, kinesiophobia, knee joint proprioception, muscle strength, and joint instability. Although no statistically significant between group differences were identified in measured characteristics, the MCID analysis indicated different patterns of clinical improvement between interventions. Aquatic CKC exercise showed greater clinically meaningful reduction in pain, WOMAC scores, and kinesiophobia, whereas land based CKC exercise demonstrated greater clinical improvements in functional mobility, proprioception, and muscle strength.

Despite the different exercise environments, no significant between‐group differences were observed for pain and joint proprioception outcomes. This finding may suggest that the observed improvements were primarily related to the characteristics of the closed kinetic chain (CKC) exercise protocol rather than the environment in which the exercises were performed. CKC exercises involve multi‐joint functional movements that increase joint compression, improve joint congruence, and reduce shear forces across the knee joint. These mechanical characteristics may stimulate articular mechanoreceptors and enhance proprioceptive input, which in turn may improve neuromuscular control and dynamic joint stability [13, 35, 36]. Furthermore, CKC exercises require coordinated activation of agonist and antagonist muscle groups and involve concentric, eccentric, and isometric muscle contractions throughout the kinetic chain. Such neuromuscular activation patterns may contribute to improved muscle strength and joint stabilization, thereby reducing excessive joint loading and mechanical stress on the knee joint. These adaptations may partly explain the reduction in pain and the improvement in proprioceptive performance observed in both groups [37]. Previous evidence also supports the relationship between pain and impaired knee proprioception in patients with knee osteoarthritis. Reduced proprioceptive accuracy has been reported in individuals with KOA and may contribute to functional limitations and progression of pain. Importantly, therapeutic exercise has been shown to improve proprioceptive accuracy in this population [38]. Similarly, Gbiri et al. reported that closed kinetic chain exercises significantly improved proprioception and muscle performance in individuals with KOA. The findings of the present study are consistent with this evidence, suggesting that CKC exercises may improve both pain and proprioceptive function, regardless of whether they are performed in aquatic or land‐based environments [39].

Additionally, Participants demonstrated significant improvement following 8 weeks of CKC exercise in both aquatic and land‐based environments except for the 40‐meter walking test, which demonstrated greater improvement in the land‐based group. The observed improvements in functional capacity may be explained by the biomechanical and neuromuscular characteristics of CKC exercises. These exercises involve multi‐joint, weight‐bearing movements that resemble daily functional activities such as walking, stair climbing, and sit‐to‐stand tasks. By promoting coordinated activation of multiple muscle groups, improving neuromuscular control, and enhancing dynamic joint stability, CKC exercises may facilitate more efficient movement patterns and improve overall functional performance [35]. The superior performance of the land‐based group in the 40‐meter walking test may be attributed to greater task‐specificity of land‐based training for gait‐related activities. Walking in a terrestrial environment requires higher ground reaction forces, greater balance demands, and more precise motor control compared to aquatic conditions, which may have better prepared participants for overground walking tasks. In contrast, aquatic exercise, while reducing joint loading and pain, may provide less specific stimulation for gait speed and endurance, potentially explaining the observed difference in this outcome. However, as between‐group differences were not observed for most other outcomes, this finding should be interpreted with caution. The present findings are consistent with previous studies reporting beneficial effects of CKC exercises on functional capacity in individuals with knee osteoarthritis. Özüdoğru and Gelecek reported comparable improvements in functional capacity following CKC and OKC exercises and suggested CKC exercise as a safe and effective intervention for patients with KOA [1]. Similarly, a systematic review demonstrated that CKC exercises such as mini‐squats, lunges, quadriceps setting, and lateral step‐ups effectively improve functional ability and may be more beneficial than other exercise modalities, including Pilates, tai chi, and resistance training [40]. Adegoke et al. also reported that both CKC and OKC exercises significantly improved pain, functional capacity, and range of motion over time, with no significant differences between exercise modalities [19]. Overall, these findings support the use of CKC exercise as an effective approach for improving functional capacity in patients with knee osteoarthritis, with both aquatic and land‐based environments providing clinically meaningful benefits. The absence of significant differences between groups for most functional outcomes further suggests that the functional gains are primarily related to the CKC exercise protocol itself rather than the exercise environment.

This study also evaluated kinesiophobia following 8 weeks of CKC exercise in aquatic and land‐based environments. Significant decrease in kinesiophobia were observed in both groups; however, no significant between‐group differences were identified. These findings suggest that CKC exercise may effectively reduce fear of movement in individuals with knee osteoarthritis, regardless of the exercise environment. Kinesiophobia is a common psychological barrier in patients with KOA and is characterized by excessive fear of movement due to concerns about pain, reinjury, or increased joint damage. Fear‐avoidance behaviors may lead to reduced physical activity, progressive muscle weakness, impaired functional performance, and further disability. Therefore, reducing kinesiophobia is considered an important component of rehabilitation in individuals with KOA [41]. The decrease in kinesiophobia observed in the present study may be explained by several interrelated mechanisms. First, reductions in pain following CKC exercise may increase patients' confidence in performing daily activities and decrease fear associated with movement. Second, improvements in muscle strength, proprioceptive input, and dynamic joint stability may enhance perceived knee control and movement safety, thereby reducing movement‐related anxiety. In addition, the functional and task‐oriented nature of CKC exercises may gradually expose patients to previously avoided activities, contributing to improved self‐efficacy and reduced fear‐avoidance behaviors [42]. The absence of significant differences between aquatic and land‐based environments may indicate that the psychological benefits of exercise are more strongly related to participation in structured therapeutic movement itself rather than the specific exercise setting. Although aquatic exercise may reduce pain through buoyancy‐related unloading of the joints, both interventions appear to provide sufficient neuromuscular and functional stimulation to improve movement confidence. These findings are supported by previous evidence. Alshahrani et al. (2022) reported significant associations between kinesiophobia, pain severity, proprioception, and functional performance in individuals with KOA, suggesting that psychological and physical impairments are closely interconnected in this population. The findings of the present study further support this relationship, as improvements in pain and physical performance following CKC exercise were accompanied by reductions in kinesiophobia in both groups [42].

Moreover, significant improvements in muscle strength were observed following 8 weeks of CKC exercise in both aquatic and land‐based environments, with no significant between‐group differences. The improvement in muscle strength may be related to the biomechanical and neuromuscular characteristics of CKC exercises. These exercises involve weight‐bearing, multi‐joint movements that promote co‐contraction of muscles around the knee joint, improve neuromuscular control, and enhance joint stability. In addition, repetitive functional loading may increase motor unit recruitment and neural adaptations, contributing to greater force production and movement efficiency [43]. The lack of significant differences between groups may indicate that the strengthening effects were primarily associated with the CKC exercise protocol itself rather than the exercise environment. Although aquatic exercise reduces joint loading through buoyancy, water resistance still provides sufficient muscular stimulation, while land‐based exercise may increase muscle activation through greater weight‐bearing demands. The present findings are consistent with previous studies investigating the effects of CKC exercise in individuals with KOA. Several studies have reported that CKC and OKC exercises produce comparable improvements in muscle strength and functional outcomes. However, some evidence suggests that CKC exercise may provide superior neuromuscular benefits by enhancing electromyographic activity and promoting greater neural adaptations [18, 44]. Cho et al. demonstrated that CKC exercise improved electromyographic activity across all quadriceps components, supporting the role of CKC training in enhancing muscular activation patterns [44].

Based on the MCID analysis, the land‐based CKC group demonstrated greater clinically meaningful improvements in TUG, 40‐m walking test, stair ascent–descent performance, knee proprioception, and muscle strength. In contrast, the aquatic CKC group showed greater decrease in pain intensity, WOMAC scores, kinesiophobia, and knee instability. The superior pain reduction observed in the aquatic group may be related to the thermal and mechanical properties of water, including buoyancy and hydrostatic pressure, which can decrease joint loading, muscle spasm, and pain sensitivity during movement. In addition, the supportive water environment may reduce fear of movement and facilitate exercise participation, leading to greater decrease in kinesiophobia and improvement in self‐reported function. Conversely, the land‐based CKC group demonstrated better performance in functional mobility and neuromuscular outcomes. These findings may be explained by greater weight‐bearing demands and vertical loading during land‐based exercise, which likely increase mechanoreceptor stimulation, muscle activation, and neuromuscular adaptations. The reduced gravitational loading in the aquatic environment may limit these adaptations despite providing a safer and less painful exercise condition. Therefore, although both interventions were beneficial, land‐based CKC exercise may provide greater improvements in functional performance and neuromuscular function, whereas aquatic CKC exercise may be more effective for pain reduction and psychological outcomes in individuals with KOA.

The study had several limitations that can be addressed in future studies. Due to legal and cultural restrictions, only female participants were included in this study. Therefore, the results can only be generalized to women. The relatively small sample size may have limited the statistical power to detect differences between the two exercise environments. Additionally, no non‐exercise control group receiving only conventional medication was included in this study because of the same recruitment‐related limitation. Furthermore, the participants' daily physical activity levels and dietary intake were not controlled. These limitations should be addressed in future research. Future research could compare the effects of open, closed, or combined chain exercises in aquatic and land environments.

## 5 Conclusions

Both aquatic and land‐based CKC exercises were effective in enhancing pain, functional performance, kinesiophobia, knee instability, proprioception, and muscle strength in individuals with knee osteoarthritis. Although no statistically significant between group differences were identified in measured characteristics, the MCID analysis indicated different patterns of clinical improvement between interventions. Aquatic CKC exercise showed greater clinically meaningful reduction in pain, WOMAC scores, and kinesiophobia, whereas land based CKC exercise demonstrated greater clinical improvements in functional mobility, proprioception, and muscle strength. These findings suggest that the rehabilitation environment may influence specific clinical outcomes despite similar overall effectiveness. Aquatic CKC exercise may be preferable for individuals with greater pain intensity or fear of movement, while land‐based CKC exercise may provide additional benefits for neuromuscular and functional performance. Future studies with larger sample sizes, longer follow‐up periods, and assessment of long‐term clinical responsiveness are recommended to better determine the comparative effectiveness of land‐based and aquatic CKC exercise programs in individuals with knee osteoarthritis.

## Author Contributions

**Mahshid Mohammadi:** conceptualizatio, data curation, investigation, project administration, writing – review and editing. **Farzaneh Gandomi:** conceptualization, methodology, formal analysis, supervision, writing – original draft, writing – review and editing. **Parviz Soufivand:** methodology, writing – review and editing, conceptualization. **Shirin Assar:** conceptualization, writing – review and editing, investigation.

## Funding

The authors have nothing to report.

## Ethics Statement

IR.RAZI.REC.1402.037

## Conflicts of Interest

The authors declare no conflicts of interest.

## Policy on Using ChatGPT and Similar AI Tools

During the preparation of this manuscript, the authors used GapGPT‐5.4 tools for the purpose of language editing and improving the readability of the text. After using this service, the authors reviewed and edited the content as needed and take full responsibility for the final version of the manuscript.

## Transparency Statement

Dr. Farzaneh Gandomi affirms that this manuscript is an honest, accurate, and transparent account of the study being reported; that no important aspects of the study have been omitted; and that any discrepancies from the study as planned (and, if relevant, registered) have been explained. She had full access to all of the data in this study and takes complete responsibility for the integrity of the data and the accuracy of the data analysis.

## Data Availability

The data that support the findings of this study are available on request from the corresponding author. The data are not publicly available due to privacy or ethical restrictions. The data that support the findings of this study are available from thecorresponding author upon reasonable request.

## Appendix 1 Closed kinetic chain exercises protocol

**Table:**

Weeks	Exercises	Purpose	Explain	Set/rep
**First**	Quadriceps setting Heel raise Sit‐standing on a chair Minin‐squat	Quadriceps, hamstring, gastrocnemius, gluteal and hip muscle strengthening	In quadriceps setting exercise, muscles while sitting on the chair, the knees should be stretched and the heels should be placed on the floor. In the mini squat, the knee position is at an angle of 30 to 45 degrees.	3/10
**Second**	Quadriceps setting Heel raise Minin‐squat Forward‐Step‐up and step‐down	Quadriceps, hamstring, gastrocnemius, gluteal muscles, hip flexors strengthening	When going up and down the stairs, the trunk should be completely straight.	3/10
**Third**	Quadriceps setting Heel raise 10RM‐Minin‐squat Forward and backward‐Step‐up and step‐down	Quadriceps, hamstring, gastrocnemius, gluteal muscles, hip flexors strengthening	In quadriceps setting exercise, muscles while sitting on the chair, the knees should be stretched and the heels should be placed on the floor. In the mini squat, the knee position is at an angle of 30 to 45 degrees. When going up and down the stairs, the trunk should be completely straight.	3/10
**Forth**	Quadriceps setting Heel raise 10RM‐Minin‐squat Lateral‐Step‐up and step‐down Wall sliding	Quadriceps, hamstring, calf muscles, gluteal muscles, hip flexors and core strengthening	In quadriceps setting exercise, muscles while sitting on the chair, the knees should be stretched and the heels should be placed on the floor. In the mini squat, the knee position is at an angle of 30 to 45 degrees. When going up and down the stairs, the trunk should be completely straight. When sliding on the wall knee flexed up to 45 degrees	3/12
**Fifth**	Quadriceps setting Heel raise 10RM‐Minin‐squat Lateral‐Step‐up and step‐down Wall sliding Forward lung	Quadriceps, hamstring, calf muscles, gluteal muscles, hip flexors and core strengthening	In quadriceps setting exercise, muscles while sitting on the chair, the knees should be stretched and the heels should be placed on the floor. In the mini squat, the knee position is at an angle of 30 to 45 degrees. When going up and down the stairs, the trunk should be completely straight. When sliding on the wall and doing lung exercises knee flexed up to 45 degrees	3/12
**Sixth**	Quadriceps setting Heel raise 10RM‐Minin‐squat Wall sliding Lateral lung	Quadriceps, hamstring, calf muscles, gluteal muscles, hip flexors and core strengthening	In quadriceps setting exercise, muscles while sitting on the chair, the knees should be stretched and the heels should be placed on the floor. In the mini squat, the knee position is at an angle of 30 to 45 degrees. When going up and down the stairs, the trunk should be completely straight. When sliding on the wall and doing lung exercises knee flexed up to 45 degrees	3/12
**Seventh**	Quadriceps setting Heel raise 10RM‐Minin‐squat Wall sliding Forward and lateral Lateral lung Single leg squat	Quadriceps, hamstring, calf muscles, gluteal muscles, hip flexors and core strengthening	In quadriceps setting exercise, muscles while sitting on the chair, the knees should be stretched and the heels should be placed on the floor. In the mini squat, the knee position is at an angle of 30 to 45 degrees. When going up and down the stairs, the trunk should be completely straight. When sliding on the wall and doing lung exercises knee flexed up to 45 degrees In single leg squat used two bars for balance	3/15
**Eight**	Quadriceps setting Heel raise 10RM‐Minin‐squat Wall sliding Forward and lateral Lateral lung Single leg squat Knee extension at standing with Pilates ball behind the knee between wall and knee	Quadriceps, hamstring, calf muscles, gluteal muscles, hip flexors and core strengthening	In the mini squat, the knee position is at an angle of 30 to 45 degrees. When going up and down the stairs, the trunk should be completely straight. When sliding on the wall and doing lung exercises knee flexed up to 45 degrees In single leg squat used two bars for balance Knee extended from the 30‐degree knee flexion	3/15
